# Lipid Profile in Patients With Amyotrophic Lateral Sclerosis: A Systematic Review and Meta-Analysis

**DOI:** 10.3389/fneur.2020.567753

**Published:** 2020-10-15

**Authors:** Jiao Liu, Xiaoyue Luo, Xueping Chen, Huifang Shang

**Affiliations:** Department of Neurology, West China Hospital, Sichuan University, Chengdu, China

**Keywords:** amyotrophic lateral sclerosis, TC, TG, HDL, LDL, mortality

## Abstract

**Background:** Studies have investigated the lipid profile in amyotrophic lateral sclerosis (ALS), including the levels of total cholesterol (TC), triglyceride (TG), high-density lipoprotein (HDL), and higher low-density lipoprotein (LDL), and the associations with mortality of ALS, but the results were inconsistent. Therefore, we conducted this meta-analysis to systematically answer this unsolved question.

**Methods:** We searched all the related studies that probed into the association between serum lipid levels and ALS based on PubMed, EMBASE, and Cochrane library from January 1990 to July 2020. The quality of the included studies was evaluated by using the Newcastle–Ottawa Scale (NOS). All the statistical analyses of this meta-analysis were performed using the Stata version 12.0 software.

**Results:** Fourteen studies with a total of 3,291 ALS patients and 3,367 controls were included. Among them, 10 studies compared the lipid profile between ALS patients and controls. The results indicated that compared with controls, ALS patients from both Europe and Asia had lower levels of TG and HDL, but the levels of TC and LDL were higher in ALS patients from Europe. However, after systemic analyses, the altered TC level was significant only in Asian ALS patients; the differences of other lipids were not significant. Concerning the effect of lipid profile on mortality of ALS, analyses of four cohort studies showed that the levels of all lipids were not associated with overall mortality in ALS.

**Conclusion:** The results of the present study showed that Asian ALS patients had lower TC levels than controls, and the levels of all lipids were not associated with mortality of ALS.

## Introduction

Amyotrophic lateral sclerosis (ALS) is a progressive and heterogeneous disease characterized by the degeneration of the upper and lower motor neurons, leading to muscle atrophy, paralysis, and death within 3–5 years (1). The pathogenesis of ALS is unknown; gene, lifestyle, and environmental factors may be involved (2). Recently, dyslipidemia was considered as an essential component of the pathological process of ALS (3), but the spectrum of lipid changes was controversial. Dupuis et al. (4) found higher levels of total cholesterol (TC) and low-density lipoprotein (LDL) in 369 ALS patients than those in 286 healthy controls (HCs). Chiò et al. (5) showed that the differences in TC, LDL, triglyceride (TG), and high-density lipoprotein (HDL) levels were not significant between 658 ALS patients and 658 HCs. Recently, Delaye et al. (6) examined the lipid profile in 30 ALS patients and 30 HCs, and they found that ALS patients had higher TC, HDL, and LDL levels than HCs. Another study showed higher TG levels and lower HDL levels in 96 patients with ALS–frontotemporal degeneration (FTD) than those in 32 HCs (7). Our previous study reported that levels of TC and HDL were significantly lower in ALS patients than in HCs (8). Moreover, the relationships between the lipids and survival of ALS have not been explored clearly. Some studies reported that hyperlipidemia was an independent predictor of survival of ALS (4, 9).

Although a previous meta-analysis had analyzed the associations of serum lipid levels with ALS, it only included six studies and did not investigate the role of lipid profile in ALS survival. In the present study, we enlarged the number of studies and conducted a deeper systematic review and meta-analysis to examine the serum lipid profile in ALS and assess the association between lipid levels and the prognosis of ALS.

## Methods

### Search Strategy

We performed a literature search within three main computerized databases, including PubMed, EMBASE, and Cochrane Library, to collect all the studies regarding the association between lipid levels and ALS from January 1990 to July 2020. The following search keywords were used: “lipid,” “cholesterol,” “triglyceride,” “low-density lipoprotein,” and “high-density lipoprotein” combined with “motor neuron^*^ disease,” “amyotrophic lateral sclerosis,” “MND,” “ALS,” or “Lou Gehrig^*^ disease.” To avoid missing literatures, we also looked through the references of all the relevant articles. Studies were screened independently by two investigators (JL and XL) using the aforementioned criteria. Any disagreements among investigators were resolved by a discussion to reach a conclusion. Our meta-analysis was registered with PROSPERO (registration number CRD42019117926).

### Selection and Exclusion Criteria

**Figure 1:**
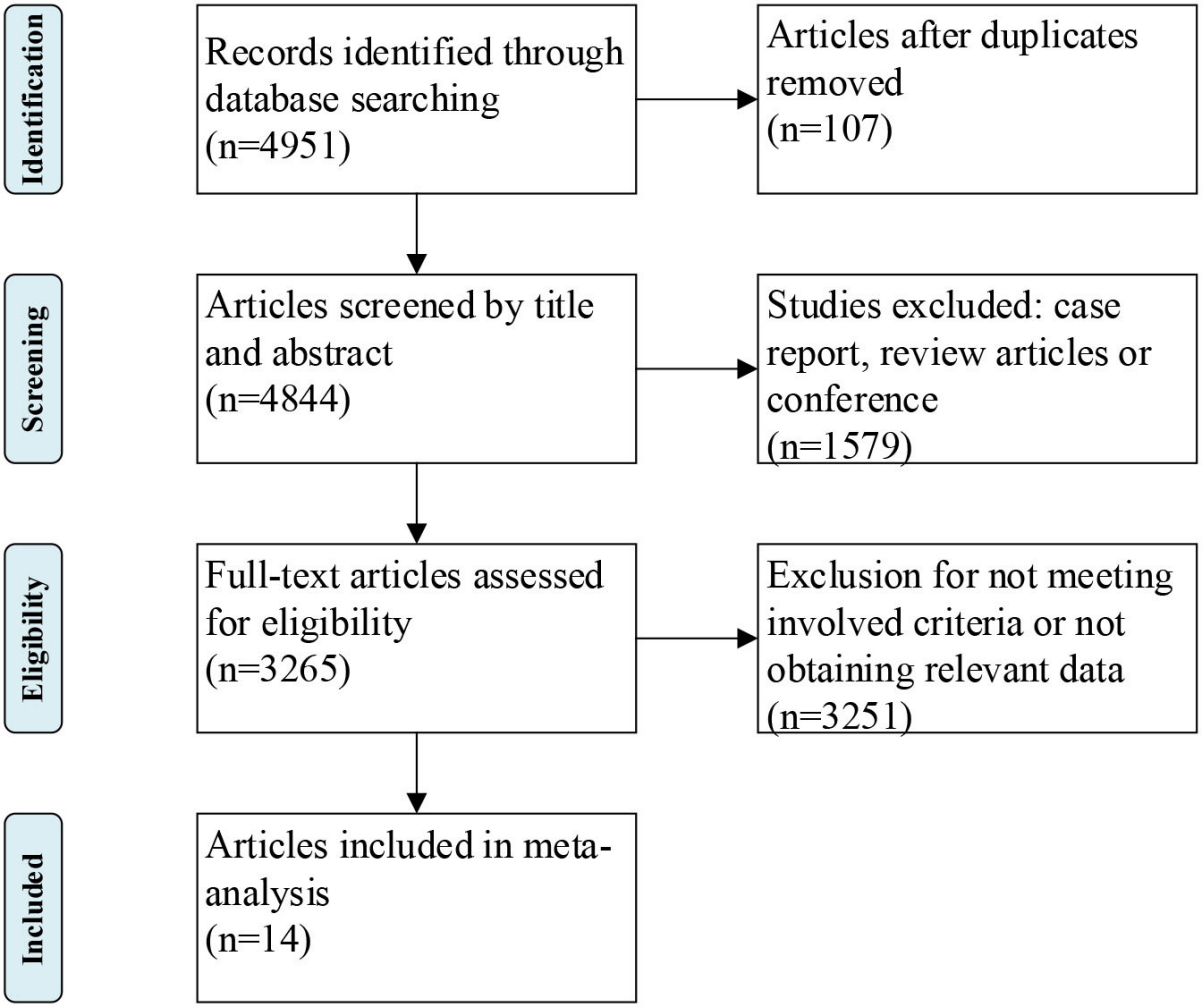
Flow chat of the study selection.

The flowchart of selection process was exhibited in Figure 1. All the included studies met the following criteria: (1) patients were diagnosed with definite or probable ALS according to the Revised El Escorial criteria or El Escorial criteria, (2) evaluated the association between serum lipid levels and ALS, (3) hazard risk (HR) with 95% confidence interval (CI) was reported in studies [odds ratio (OR) with 95% CI of one study was excluded (10)], or the mean value of serum lipid levels with standard deviation (SD) was reported in studies, and (4) published in English. The exclusion criteria were demonstrated as follows: (1) duplicate reports, (2) *in vitro* or animal studies, (3) case reports, reviews, or meta-analysis, and (4) necessary data were not available.

### Data Extraction and Quality Evaluation

The following data were extracted from each included study: first author, publication year, country of origin, sample size of ALS cases and HCs, ALS definition criterion, adjusted variables, and duration of follow-up in ALS patients. To avoid mistakes and bias, two authors extracted data separately, and a third investigator verified the data abstracted by the two authors. If the articles did not provide complete data, we sent an email to the author asking for the necessary data (11–13). Study quality was assessed by the Newcastle–Ottawa Scale (NOS) (14), and a score of 7 or above was considered to be of high quality.

### Statistical Analyses

To compare serum lipid levels in ALS patients and HCs, standardized mean difference (SMD) with 95% CI was used. To evaluate the association between serum lipid levels and all-cause mortality risk in ALS patients, the HR with 95% CI was used. TC, HDL, and LDL concentrations were converted from mmol/L to mg/dl using a ratio of 38.67 (1 mmol/L ≈ 38.67 mg/dl), and TG concentration was converted from mmol/L to mg/dl using a ratio of 88.545 (1 mmol/L ≈ 88.545 mg/dl). Heterogeneity analysis was assessed using the Cochrane Q test and *I*^2^ statistic. *I*^2^ > 50% or *P* < 0.1 represented substantial heterogeneity, and random effects model was chosen. Otherwise, fixed effects model was utilized (15). To assess the influence of each study on the pooled estimate, sensitivity analysis was applied by removing each study by turns and switching effects model. Publication bias was estimated by the funnel plot and Egger's test (16). Probability value *P* < 0.05 was considered to be statistically significant. All statistical analysis of this meta-analysis was performed using the Stata version 12.0 software.

## Results

### Search Results and Study Characteristics

The literature research identified 4,951 papers from computerized databases according to the search strategy (Figure 1). After removing the duplicate studies, case report, review, or conference, a careful additional review of the full-text was needed for the remaining 3,265 articles, and 14 studies with a total of 3,291 ALS patients and 3,367 controls met our inclusion criteria at last (4–6, 8, 9, 17–25) (Supplementary Tables 1, 2). Quality assessment was performed according to the NOS criteria. The NOS score ranged from 6 to 8 points in these studies, suggesting that these studies were of moderate to high quality.

### Lipid Profile in ALS Patients and HCs

**Figure 2:**
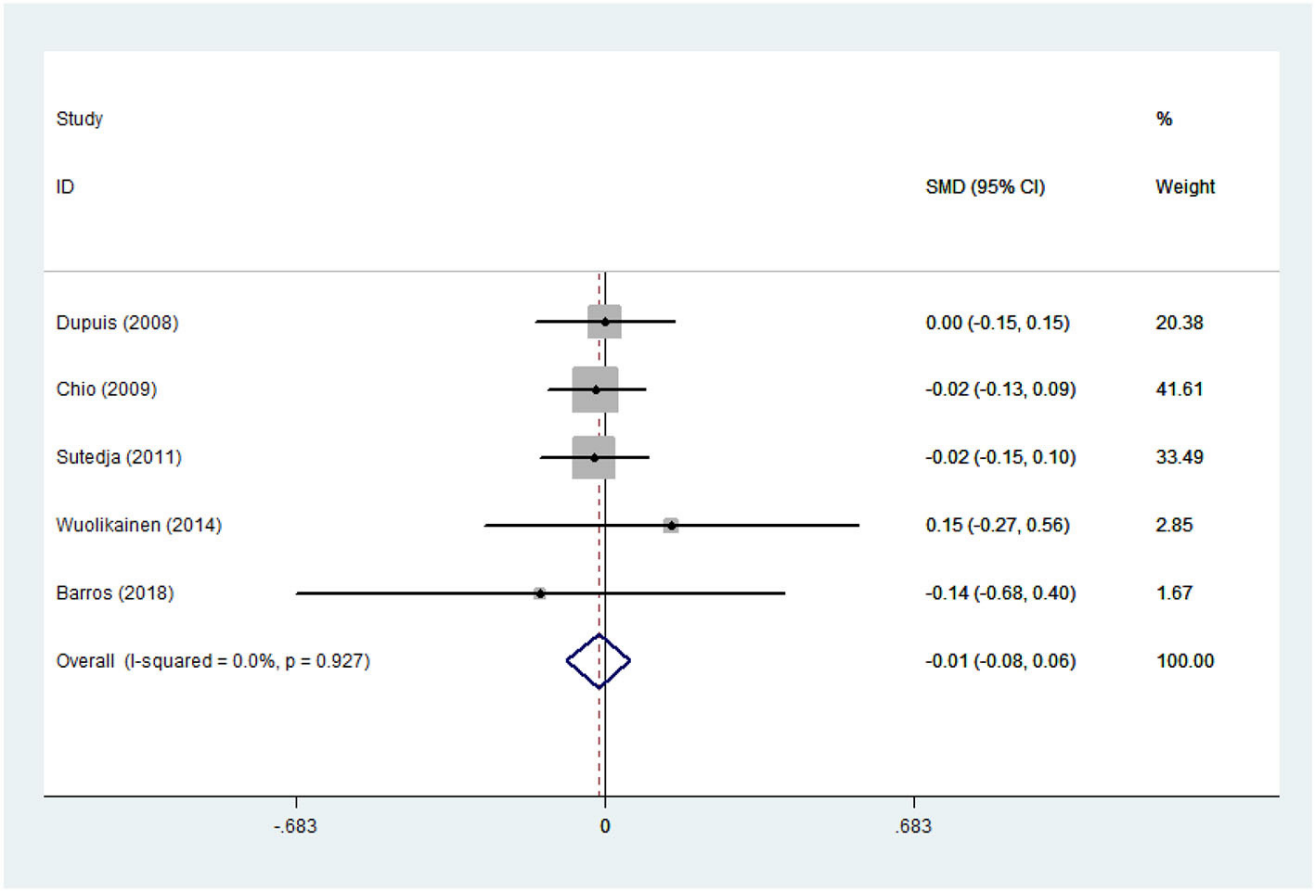
High-density lipoprotein (HDL) levels in ALS patients compared to that in controls (European).

**Figure 3:**
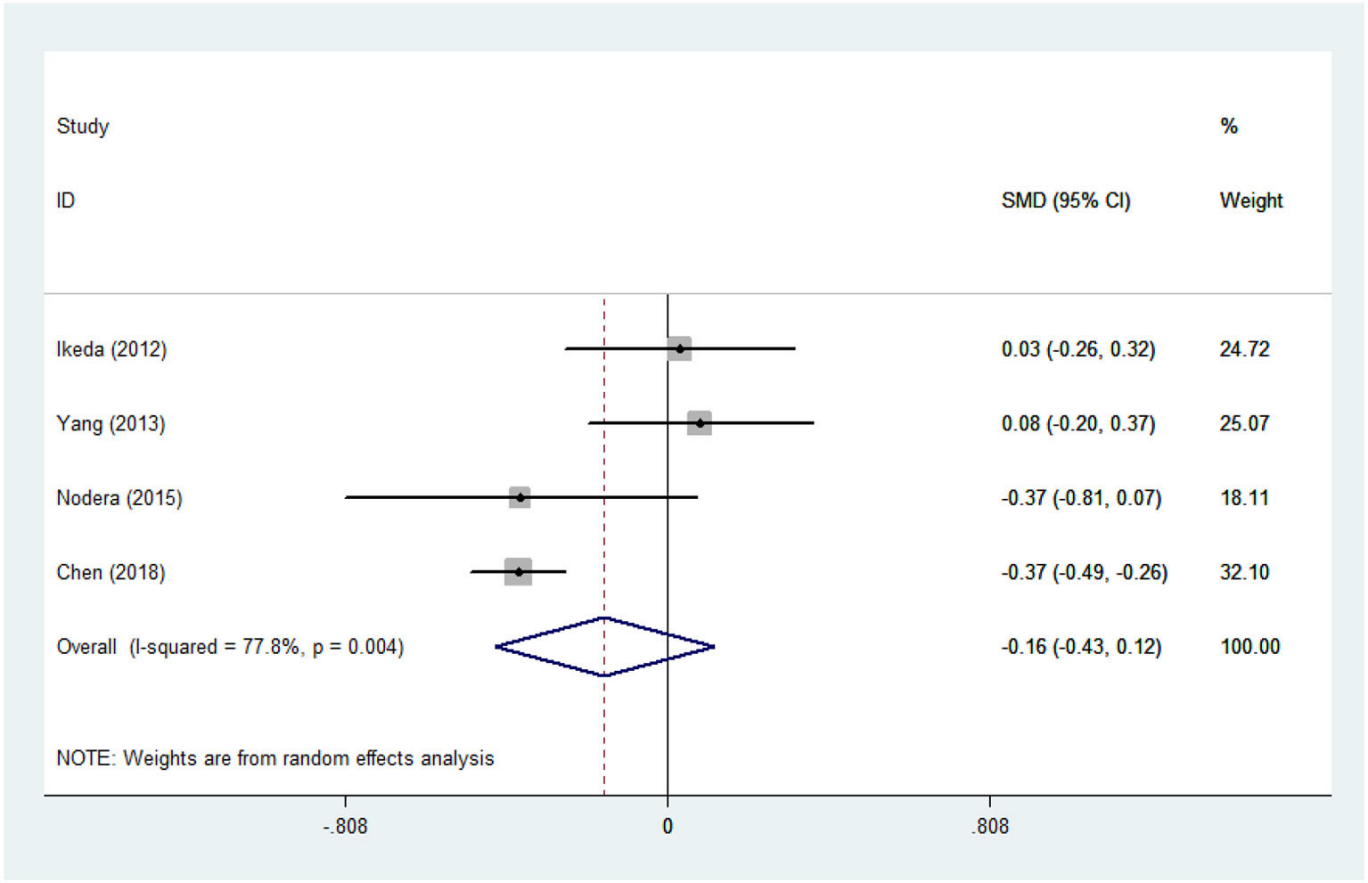
HDL levels in ALS patients compared to that in controls (Asian).

**Figure 4:**
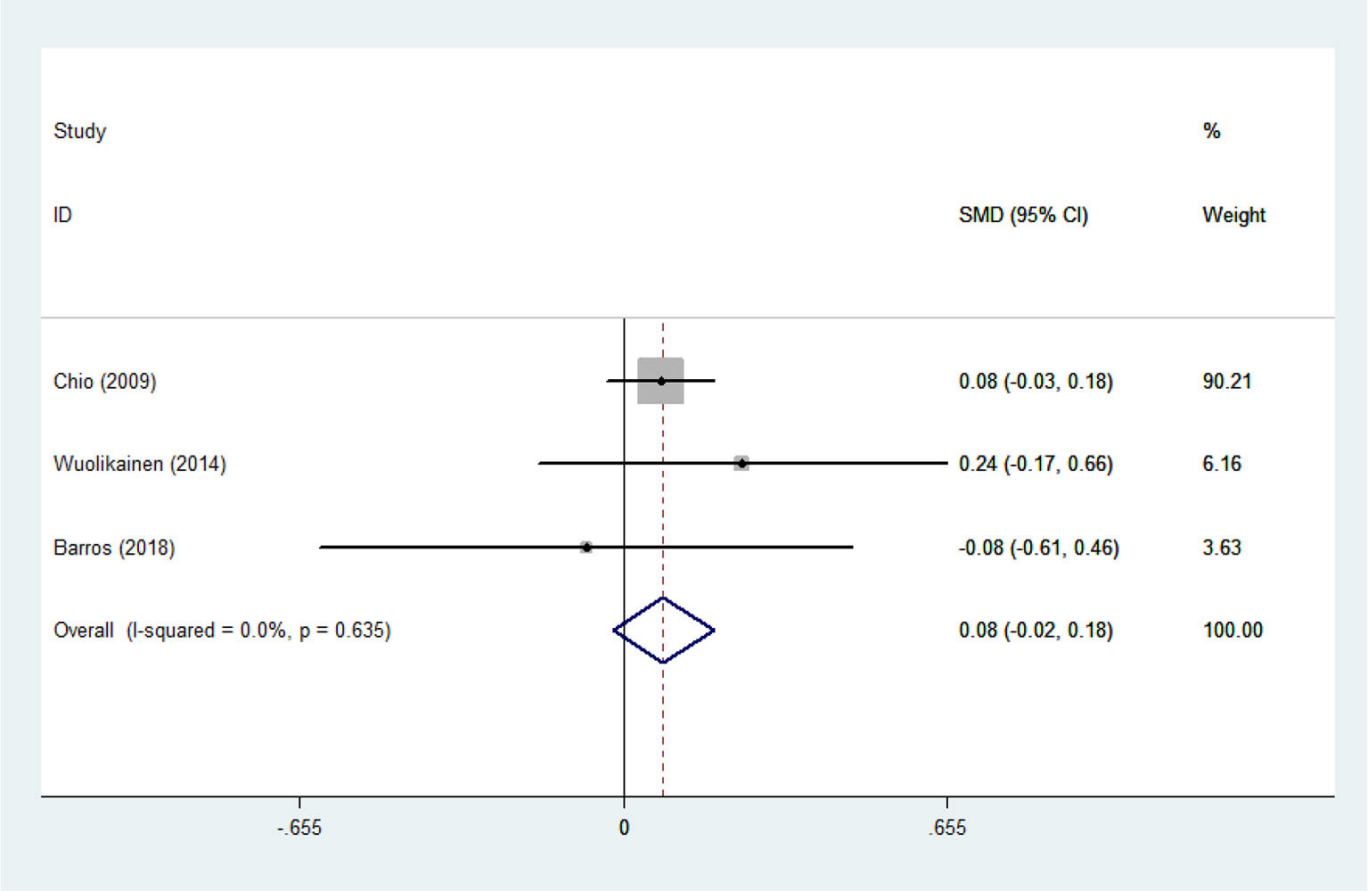
Low-density lipoprotein (LDL) levels in ALS patients compared to that in controls (European).

**Figure 5:**
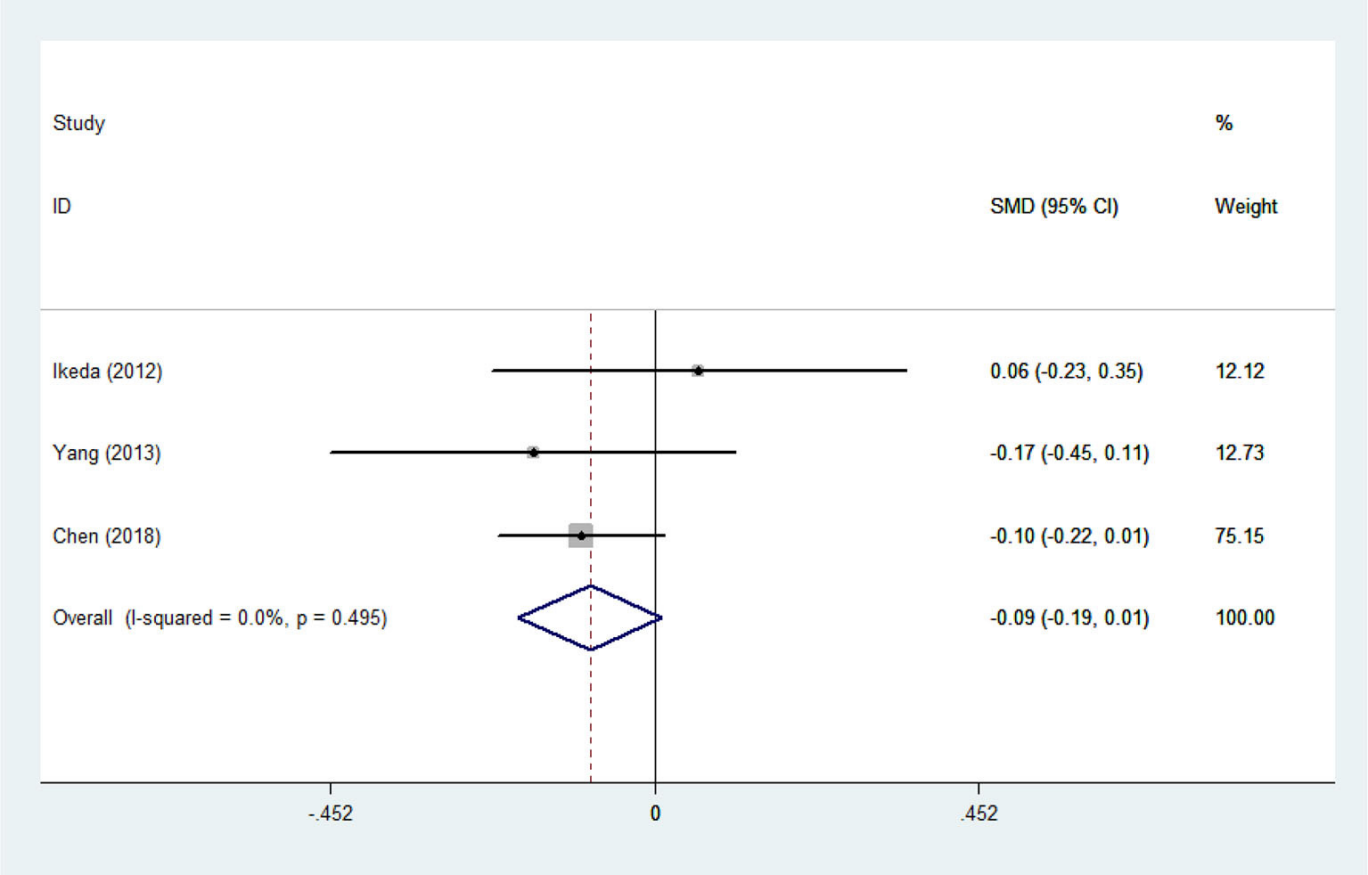
LDL levels in ALS patients compared to that in controls (Asian).

**Figure 6:**
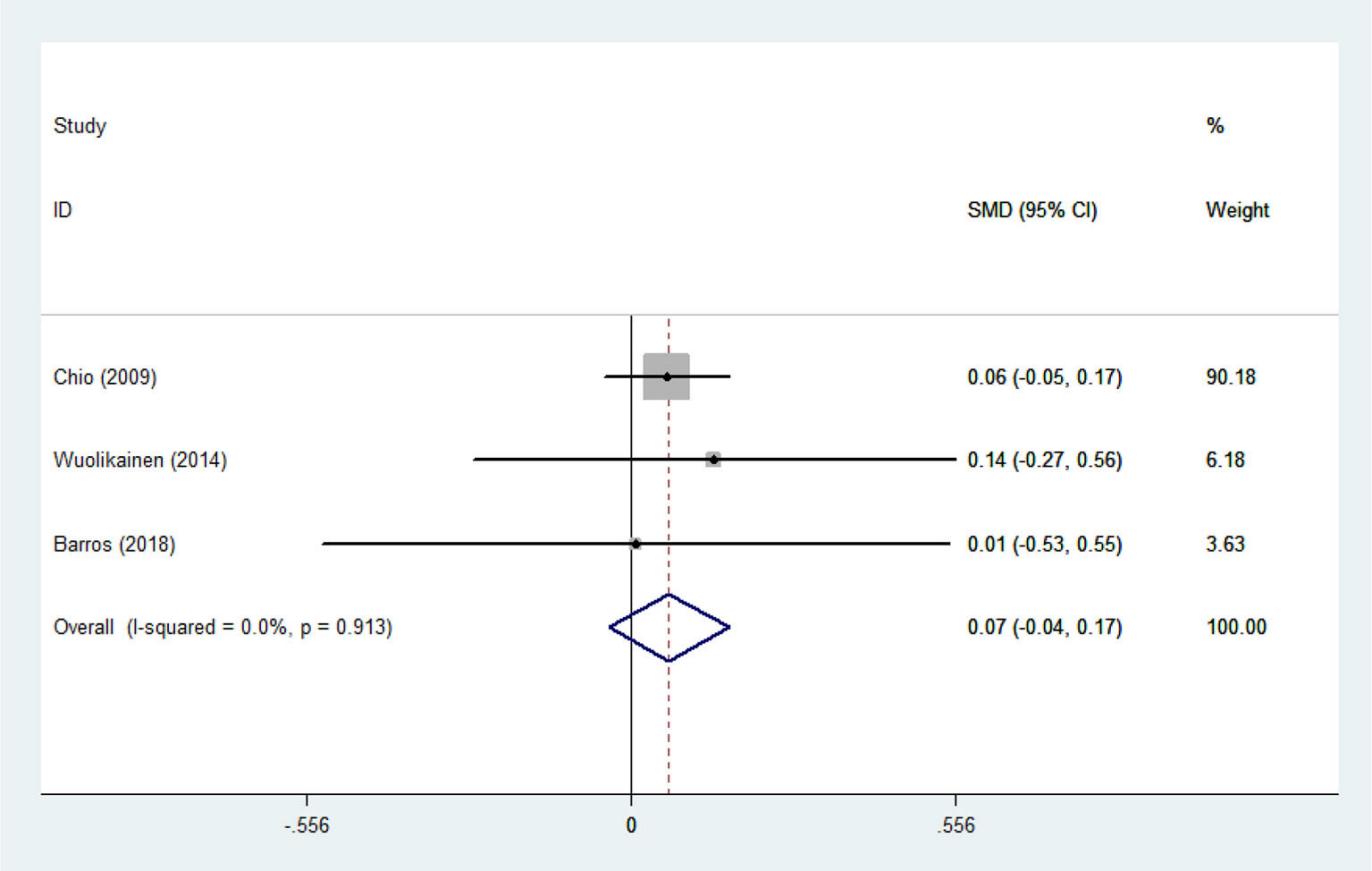
Total cholesterol (TC) levels in ALS patients compared to that in controls (European).

**Figure 7:**
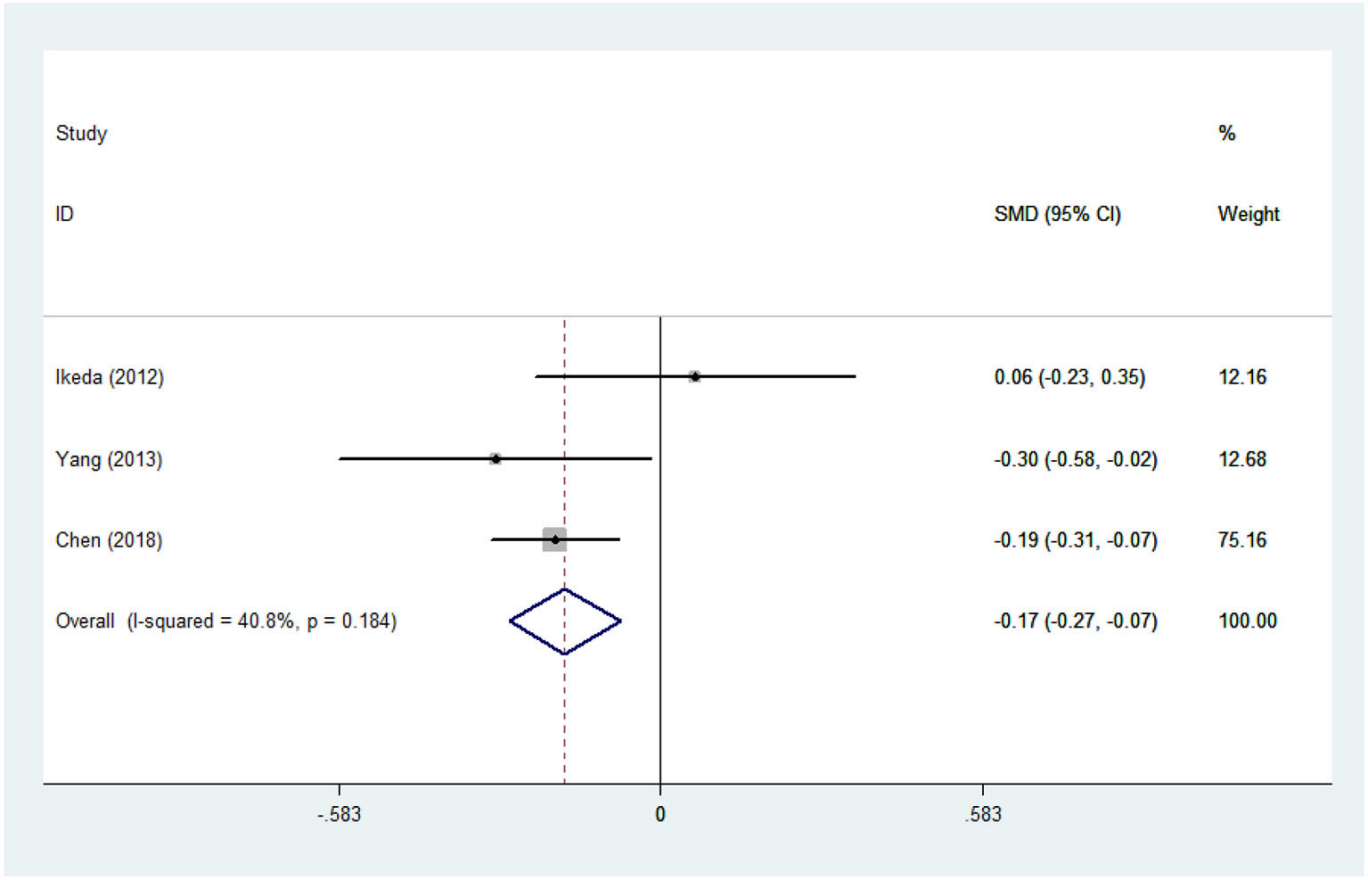
TC levels in ALS patients compared to that in controls (Asian).

**Figure 8:**
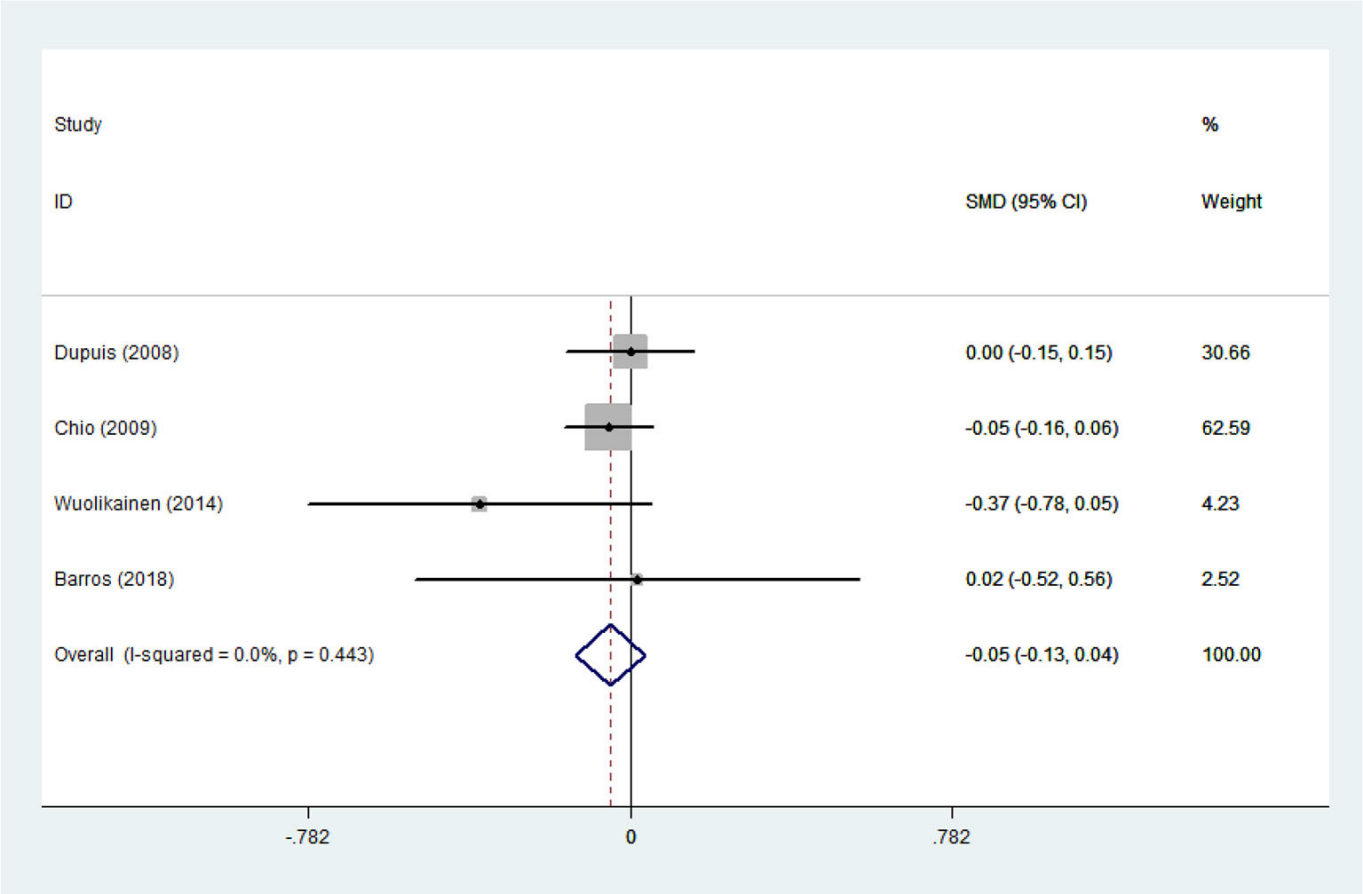
Triglyceride (TG) levels in ALS patients compared to that in controls (European).

**Figure 9:**
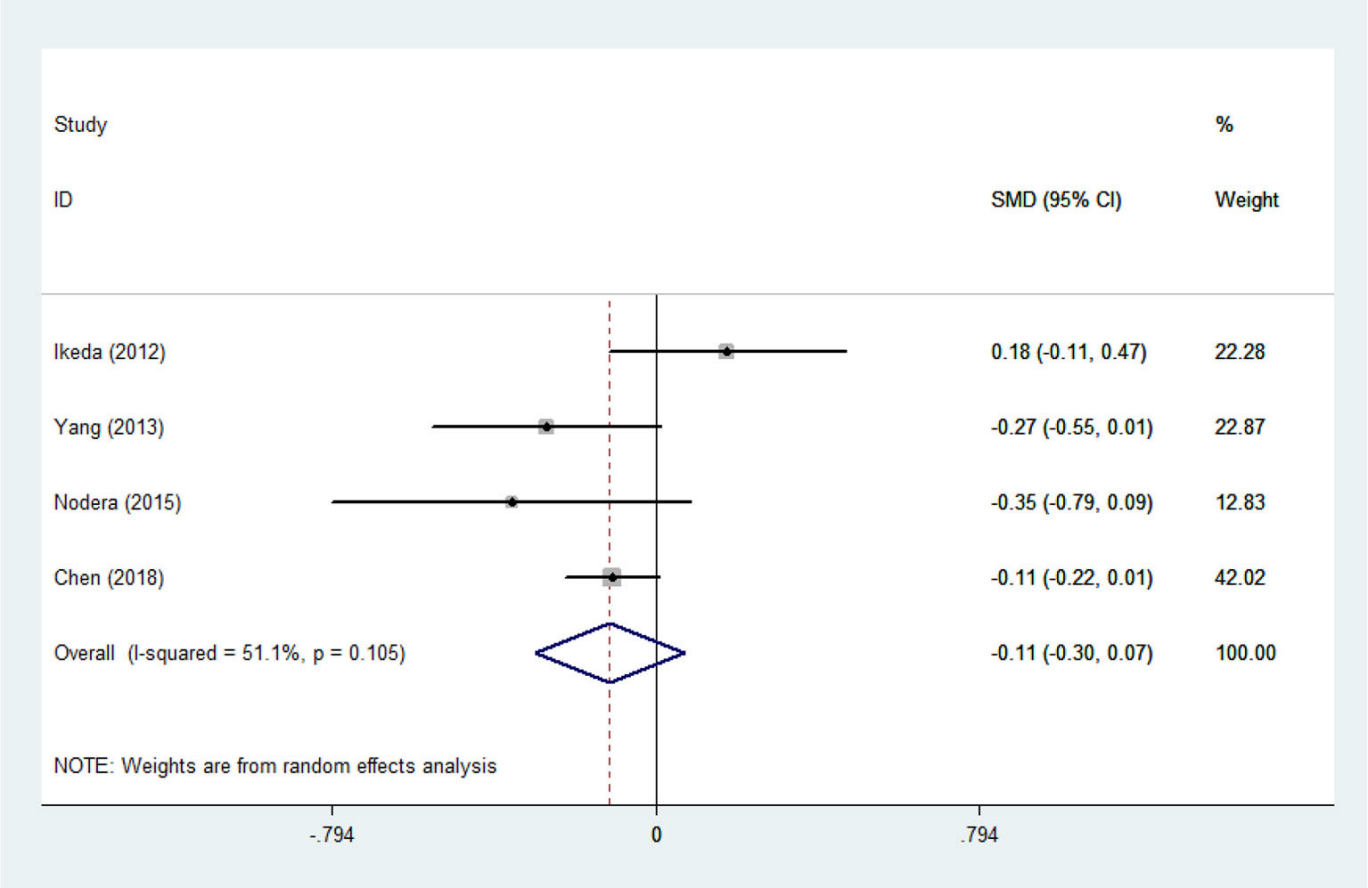
TG levels in ALS patients compared to that in controls (Asian).

There was substantial heterogeneity among those 10 studies, including studies related to HDL (*P* < 0.05, *I*^2^ = 77.8%) and TG (*P* < 0.05, *I*^2^ = 51.1%) in Asian ALS patients. Thus, a random effects model was used. Compared with the HCs, both European and Asian ALS patients had lower concentrations of TG and HDL. The TC and LDL levels were lower in Asian ALS patients, but higher in European ALS patients. However, only the decreased TC level in Asian ALS patients was significant. In addition, there is no significance when we took TC levels in both European and Asian ALS patients into consideration, and the differences of other lipids were not significant (Figures 2–9). Sensitivity analysis demonstrated that the pooled SMD was stable after omitting each study; therefore, the results were reliable (Supplementary Figures 1–8).

### Lipid Levels and Mortality Risk Among ALS Patients

**Figure 10:**
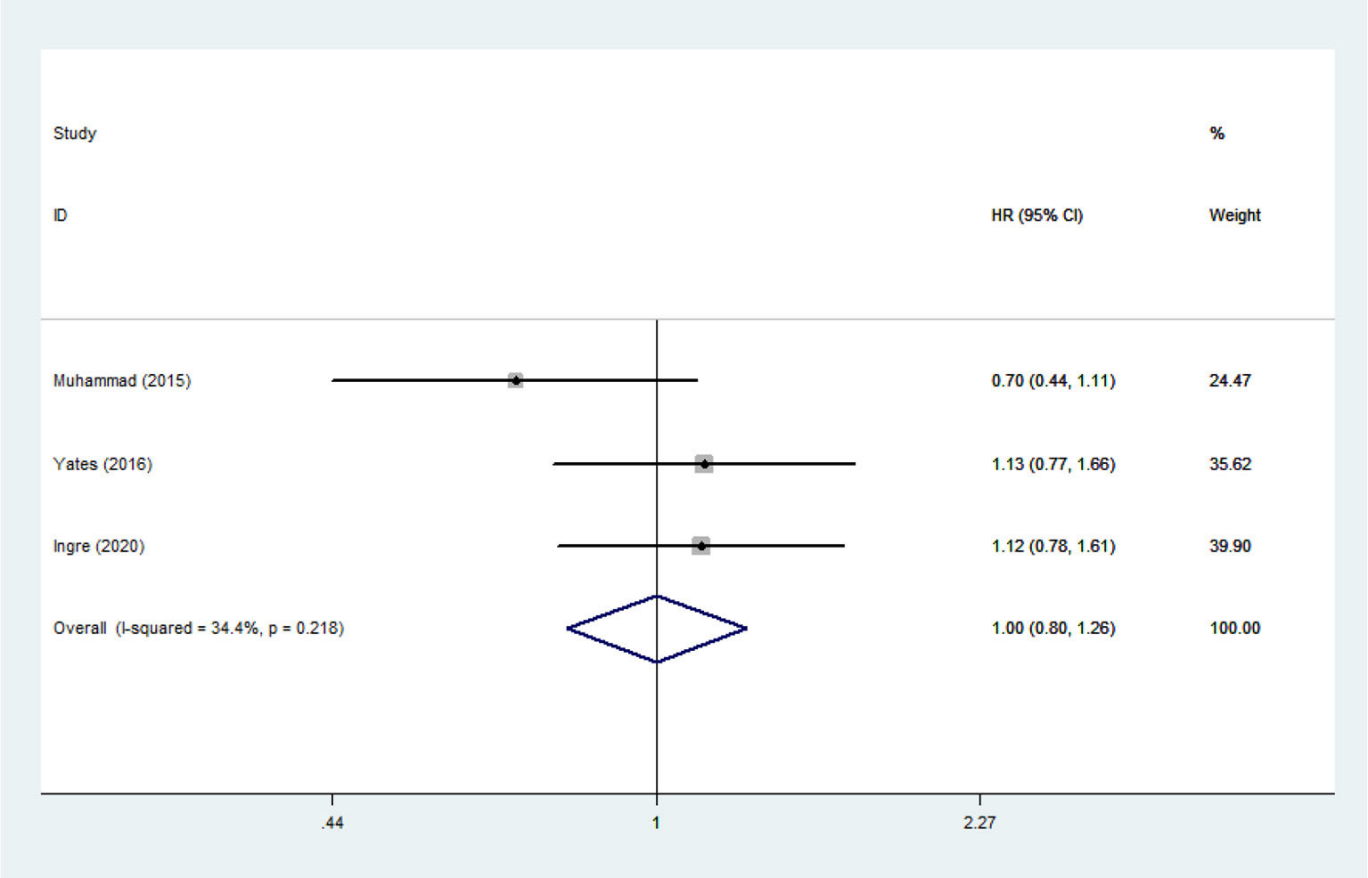
The relationship between HDL and risk of death among ALS patients.

**Figure 11:**
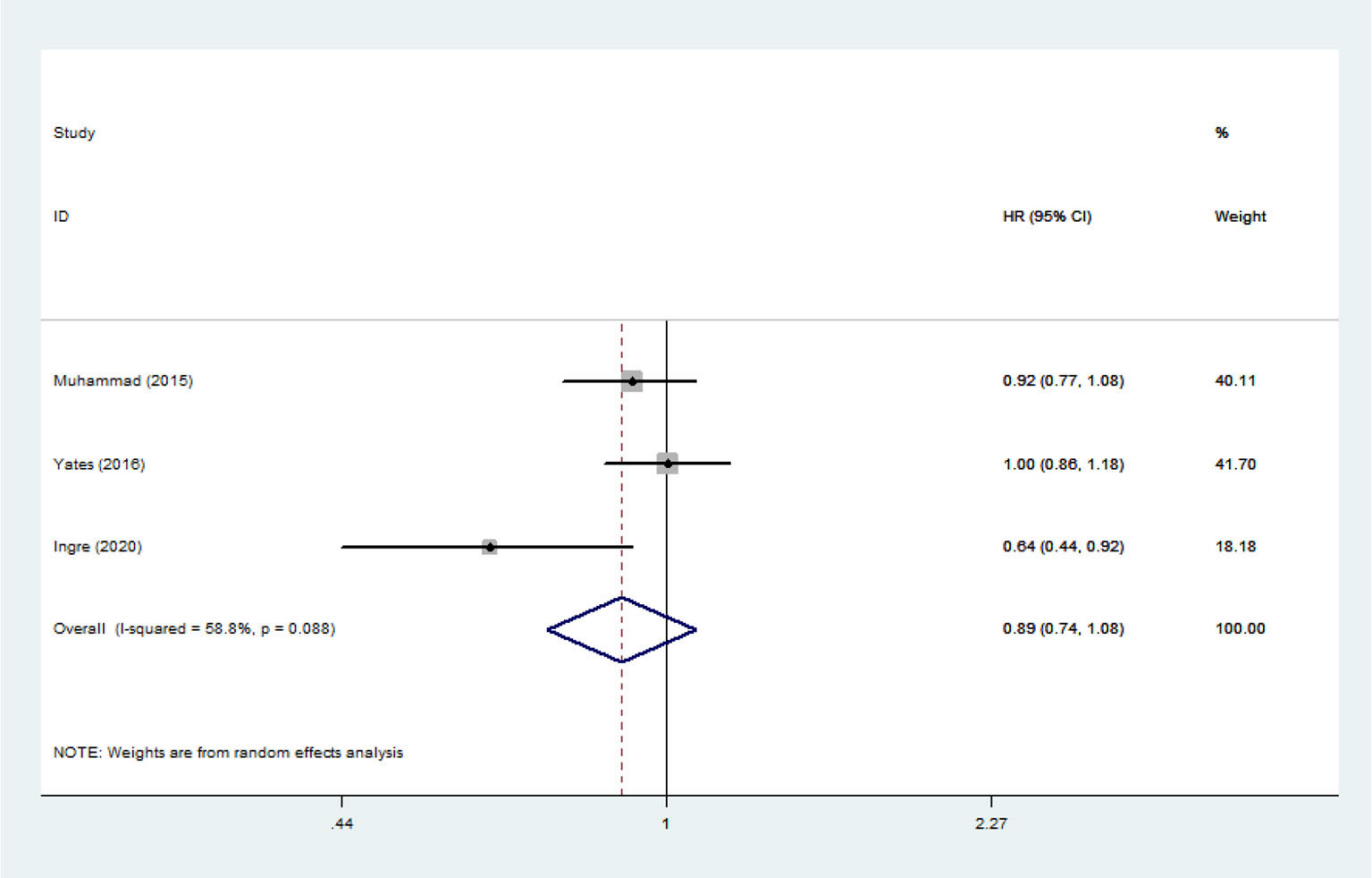
The relationship between LDL and risk of death among ALS patients.

**Figure 12:**
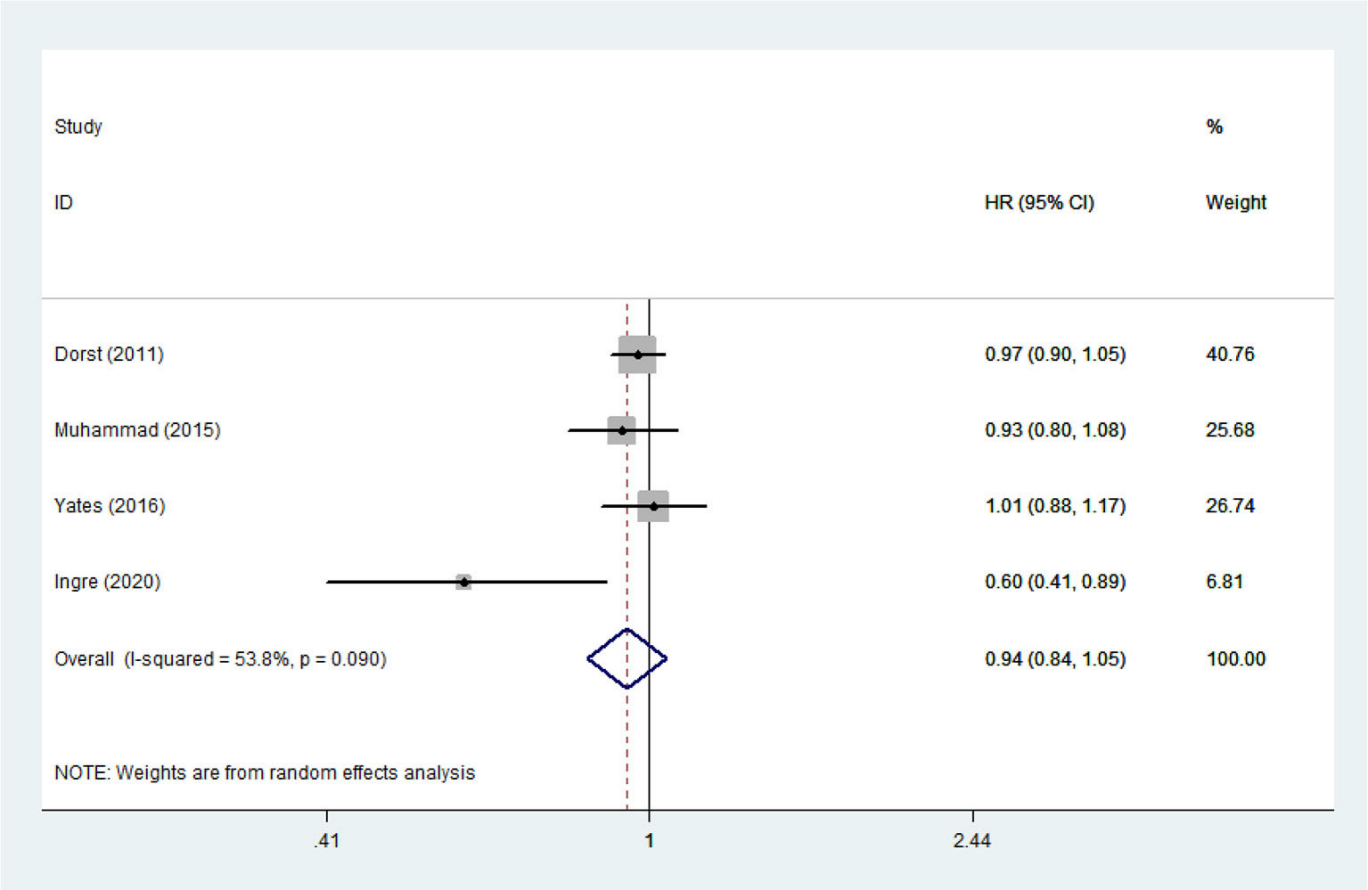
The relationship between TC and risk of death among ALS patients.

**Figure 13:**
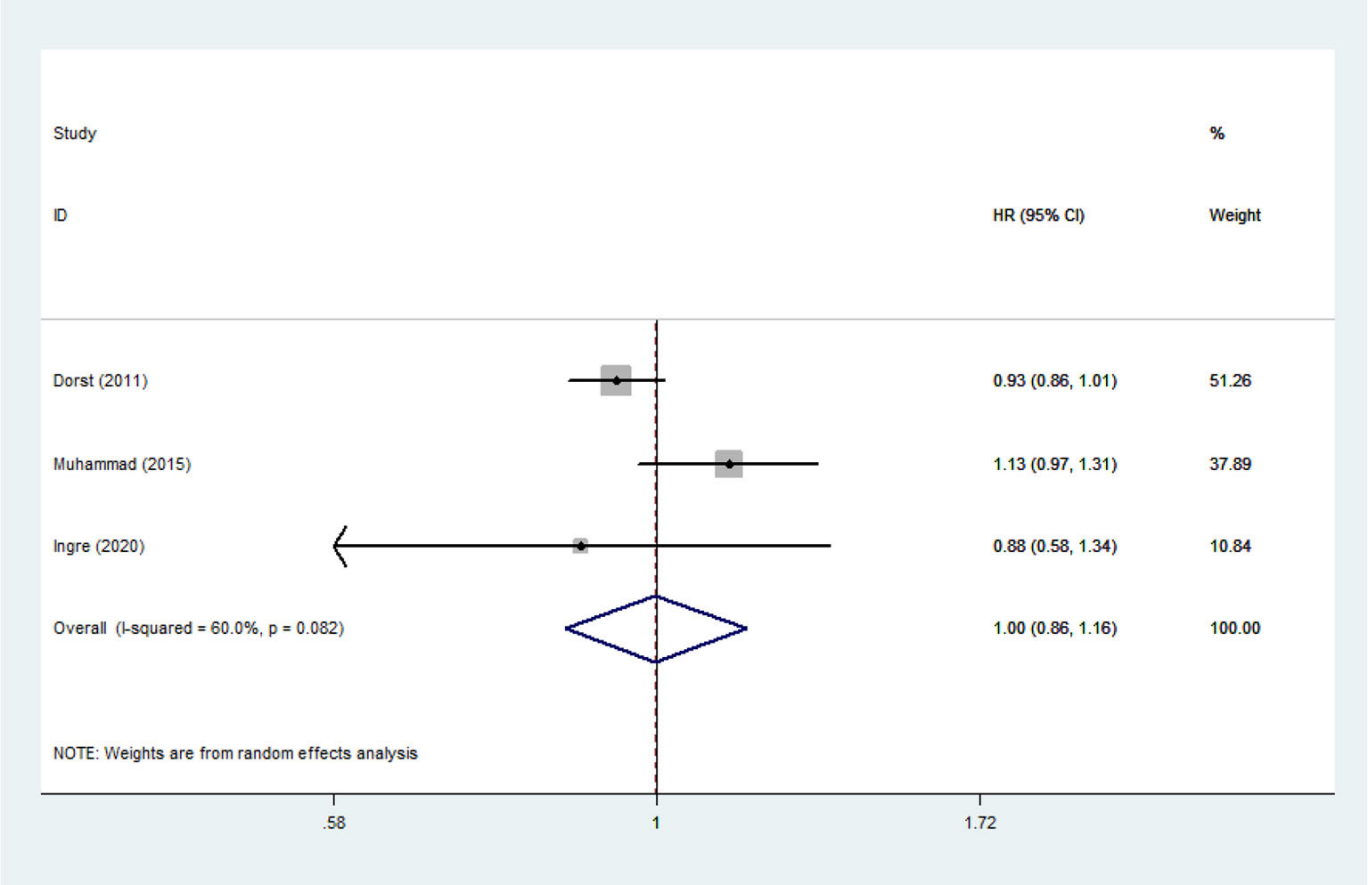
The relationship between TG and risk of death among ALS patients.

Heterogeneity analysis suggested that there was no obvious heterogeneity among those four studies, including study related to HDL (*P* > 0.05, *I*^2^ = 34.4%), and a fixed effects model was used to pool the risk estimate. The results showed that the levels of TC, TG, LDL, and HDL in ALS patients were not associated with mortality (*P* > 0.05) (Figures 10–13). Sensitivity analysis indicated that the pooled HR was stable after omitting each study.

## Discussion

A previous meta-analysis did not identify a statistical difference in the lipid levels between ALS patients and HCs (26). The inconsistent findings obtained from different studies could be caused by dietary habits, gender, and regional differences and the use of cholesterol-lowering medication. The current meta-analysis included larger number of studies, re-evaluated the lipid profile in ALS, and explored the association between serum lipid levels and the survival of ALS.

We found that the serum TC levels in Asian ALS patients were significantly lower than those in HCs. Different from the previous meta-analysis, our study explored the lipid levels between ALS patients and HCs by dividing the included studies into Asian and European groups. Moreover, it is well known that dietary habits in Asian and European groups are dramatically different, which might be relevant with our positive findings.

However, the differences between male and female were not highlighted in our study for lack of all the related data collected in the included studies. The detailed information we needed could not be obtained from the mails sent to the corresponding author. Original studies gave the whole data of TC, TG, HDL, and LDL levels in the entire population, but without the data of lipids in male and female ALS patients, we could not perform the adjust analysis for gender. One study from Asia collected serological data from 92 ALS patients and 92 age-, sex-, and body mass index-matched HCs and found significant increases of serum TC, TG, and LDL levels in female ALS patients, but not in male ALS patients (18). Conversely, another study from Asia examined the serum lipid levels of 95 patients with ALS and 99 age- and sex-matched HCs and found that TC, TG, and LDL levels in male ALS patients were significantly lower than those in HCs (19). Similarly, our previous study also found that TC levels were significantly lower in male ALS patients (8). The exact cause of gender-related differences is unclear. It could be partially explained by the predilection toward a greater prevalence of ALS among men (2), and the finding that estrogen delays disease progression in ALS mice (27).

Studies focusing on serum lipid levels and ALS survival were increasing in recent years, but the association between them was controversial. Huang et al. found that ALS patients with higher serum TG levels had a longer survival time than those with lower serum TG levels (26). Similarly, Mandrioli et al. showed that an increase level of TG was inversely associated with the odds of death or tracheostomy (10). Ahmed et al. reported that a higher TC level was correlated with 3.25 times improved survival in ALS–FTD patients (7). Some studies hold the idea that hyperlipidemia is a significant prognostic factor for the survival of patients with ALS (4, 9), but other studies demonstrated that the prognosis of ALS was not influenced by the lipid profile (5, 17, 21, 28, 29), and these findings were in accordance with our current study. However, our results should be interpreted with care because only four studies from Europe up to our standard were included in the final analysis, and many confounding factors, including areas and ethnic variations, the use of lipid-lowering drugs, and the period of delay diagnosis, among others, should not be neglected. Therefore, more high-quality longitudinal studies focusing on the relationship between serum lipids and survival in ALS are needed.

There were several unavoidable limitations in the present study. First, this meta-analysis only included studies published in English, which could inevitably lead to selection bias. Second, serum lipid levels can be affected by many factors, such as dietary habits and the use of cholesterol-lowering medication, which were not mentioned in some included studies. Third, we only included 14 studies at last because some studies did not give the completed data, though we had sent emails to the authors.

## Conclusion

In summary, our meta-analysis indicated that compared with the HCs, ALS patients from both Europe and Asia had lower TG and HDL levels, and ALS patients from Europe had higher levels of TC and LDL. However, only the difference in TC levels in Asian ALS patients was significant. Moreover, the overall survival of ALS patients was uncorrelated with the levels of TC, TG, LDL, and HDL. Therefore, meticulous designed randomized comparison studies with longitudinal follow-up are required to verify the exact role of lipid profile in ALS.

## Data Availability Statement

All datasets presented in this study are included in the article/Supplementary Material.

## Author Contributions

XC and HS conceived this study. JL and XL did the literature review and the statistical analysis. JL wrote the manuscript while all the authors revised and discussed the final edition.

## Conflict of Interest

The authors declare that the research was conducted in the absence of any commercial or financial relationships that could be construed as a potential conflict of interest.
