# Supplementary material for: Lipid Profile in Patients With Amyotrophic Lateral Sclerosis: A Systematic Review and Meta-Analysis
Source: Front Neurol. 2020 Oct 15;11:567753. doi: 10.3389/fneur.2020.567753 (PMC7593382; doi:10.3389/fneur.2020.567753)
Supplement: Supplementary file 1 [file Data_Sheet_1.docx]

Table S1. Characteristics of the included ten studies

| Study | location | Participants (cases/controls) | Serum lipid levels (ALS) | | |  | NOS score |
| --- | --- | --- | --- | --- | --- | --- | --- |
|  |  |  | HDL  (mg/dl) | LDL  (mg/dl) | TC  (mg/dl) | TG  (mg/dl) |  |
| Dupuis2008 | France | 369/286 | 60±150 | 160±40 | 250±50 | 130±90 | 7 |
| Chio2009 | Italy | 658/658 | 59.3±15.5 | 128.7±38.6 | 211.1±44.1 | 115.1±54.6 | 7 |
| Sutedja2011 | Netherlands | 303/2100 | 59.3±18.22 | 123.17±37.07 | 211.52±41.38 | ---- | 8 |
| Ikeda2012 | Japan | 92/92 | 57.3±15.52 | 126.8±73.03 | 207.61±37.19 | 128.04±50.49 | 8 |
| Yang2013 | Korea | 95/99 | 47.28±12.14 | 115.56±28.72 | 188.17±33.93 | 126.73±73.86 | 7 |
| Wuolikainen2014 | Sweden | 52/40 | 70.54±21.32 | 131.27±34.36 | 230.86±52.59 | 97.4±39.85 | 7 |
| Nodera2015 | Japan | 54/37 | 54.3±13.5 | 118.5±30.8 | ---- | 150.3±70.7 | 7 |
| Delaye2017 | France | 30/29 | 60.47±28.68 | ---- | 251.74±83.53 | ---- | 7 |
| Barros2018 | Brazil | 27/26 | 43.7±9.5 | 121.3±38.1 | 190.6±45.6 | 126±55.4 | 8 |
| Chen2018 | China | 571/571 | 55.04±15.12 | 106.18±30.12 | 181.36±35.96 | 128.39±98.28 | 8 |

| TC: total cholesterol; TG: triglyceride; HDL: high-density lipoprotein, LDL: low-density lipoprotein; NOS= Newcastle-Ottawa Scale |
| --- |

Table S2. Characteristics of the included four studies

| Study | location | ALS definition | Duration of follow-up(months) | study size | adjusted variable | NOS score |
| --- | --- | --- | --- | --- | --- | --- |
| Dorst2011 | Germany | REEC | 92 | 488 | Age/site of onset, ALSFRS-R, BMI, Disease duration | 8 |
| Rafiq2015 | UK | EEC | 18 | 512 | Age, gender, site of onset, Disease duration, FVC and ALSFRS-R | 6 |
| Yates2016 | UK | EEC | 18 | 512 | Age, gender, site of onset, Disease duration, FVC and ALSFRS-R | 6 |
| Ingre2020 | Sweden | REEC | 36 | 99 | Sex, age at diagnosis, site of onset, diagnostic delay, BMI,  ALSFRS-R, and progression rate | 8 |

| ALS=Amyotrophic lateral sclerosis; EEC=EI Escorial criteria; REEC=Revised El Escorial criteria; ALSFRS-R=ALS Functional Rating Scale-Revised; BMI=Body mass index; NOS= Newcastle-Ottawa Scale |
| --- |


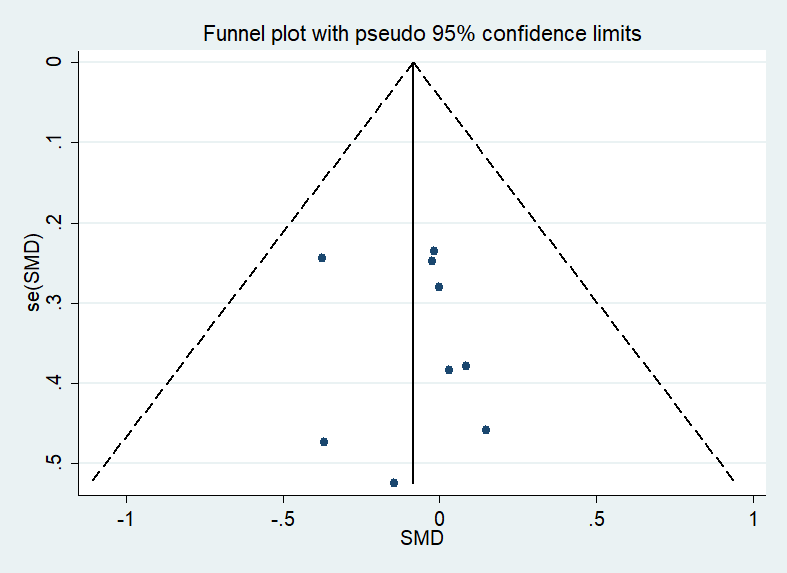


Figure S1. Publication bias funnel plots for HDL and ALS analysis.


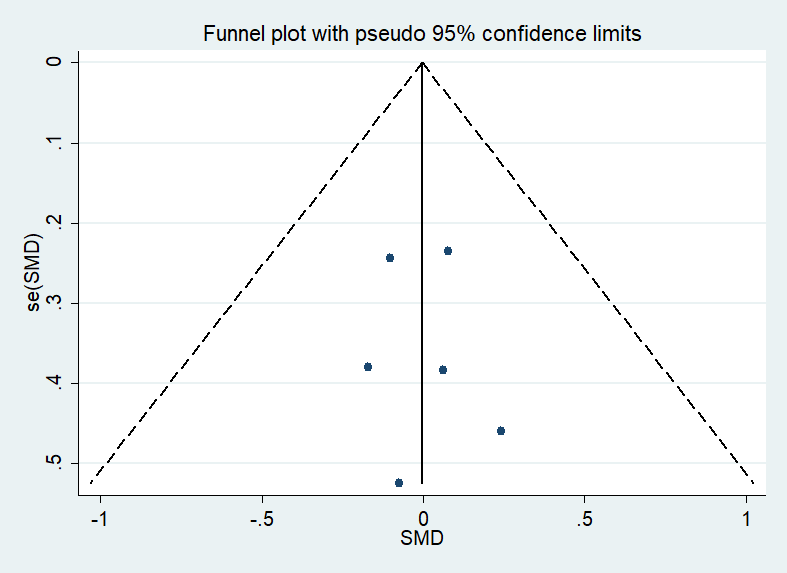


Figure S2. Publication bias funnel plots for LDL and ALS analysis.


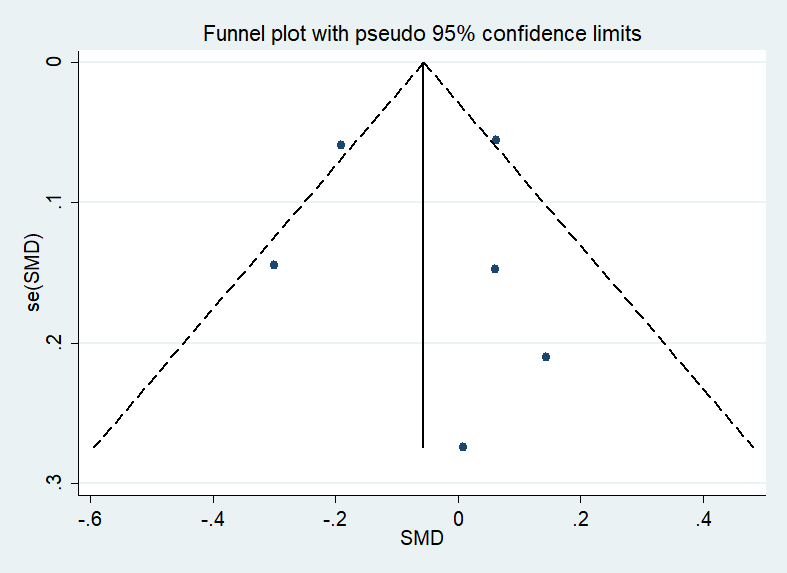


Figure S3. Publication bias funnel plots for TC and ALS analysis.


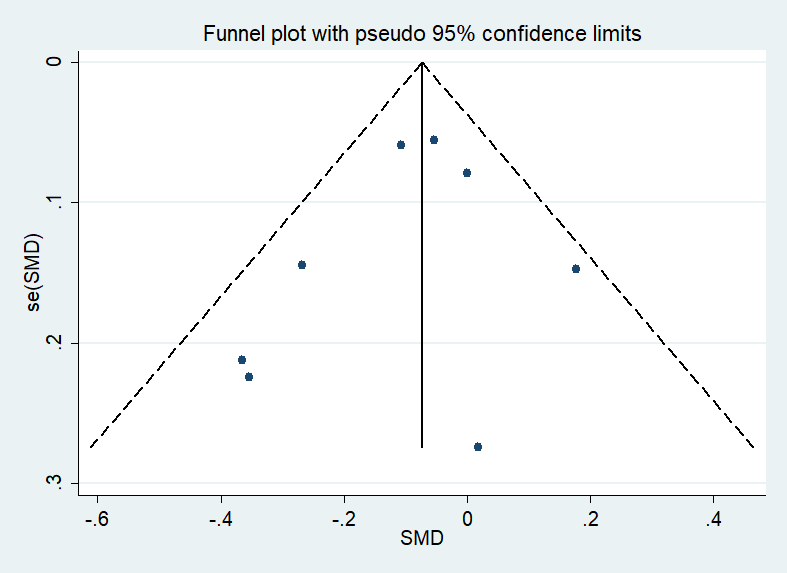


Figure S4. Publication bias funnel plots for TG and ALS analysis.

Figure S5. Egger’s publication bias plot for HDL and ALS analysis.

Figure S6. Egger’s publication bias plot for LDL and ALS analysis.

Figure S7. Egger’s publication bias plot for TC and ALS analysis.

Figure S8. Egger’s publication bias plot for TG and ALS analysis.
